# SFPQ regulates the accumulation of RNA foci and dipeptide repeat proteins from the expanded repeat mutation in *C9orf72*

**DOI:** 10.1242/jcs.256602

**Published:** 2021-02-19

**Authors:** Mirjana Malnar, Boris Rogelj

**Affiliations:** 1 Department of Biotechnology, Jožef Stefan Institute, 1000 Ljubljana, Slovenia; 2 Graduate School of Biomedicine, Faculty of Medicine, University of Ljubljana, 1000 Ljubljana, Slovenia; 3 Biomedical Research Institute, 1000 Ljubljana, Slovenia; 4 Faculty of Chemistry and Chemical Engineering, University of Ljubljana, 1000 Ljubljana, Slovenia

**Keywords:** Amyotrophic lateral sclerosis, *C9orf72*, Dipeptide repeat proteins, Frontotemporal dementia, RNA foci, SFPQ

## Abstract

The expanded GGGGCC repeat mutation in the *C9orf72* gene is the most common genetic cause of the neurodegenerative diseases amyotrophic lateral sclerosis (ALS) and frontotemporal dementia (FTD). The expansion is transcribed to sense and antisense RNA, which form RNA foci and bind cellular proteins. This mechanism of action is considered cytotoxic. Translation of the expanded RNA transcripts also leads to the accumulation of toxic dipeptide repeat proteins (DPRs). The RNA-binding protein splicing factor proline and glutamine rich (SFPQ), which is being increasingly associated with ALS and FTD pathology, binds to sense RNA foci. Here, we show that SFPQ plays an important role in the *C9orf72* mutation. Overexpression of SFPQ resulted in higher numbers of both sense and antisense RNA foci and DPRs in transfected human embryonic kidney (HEK) cells. Conversely, reduced SPFQ levels resulted in lower numbers of RNA foci and DPRs in both transfected HEK cells and *C9orf72* mutation-positive patient-derived fibroblasts and lymphoblasts. Therefore, we have revealed a role of SFPQ in regulating the *C9orf72* mutation that has implications for understanding and developing novel therapeutic targets for ALS and FTD.

This article has an associated First Person interview with the first author of the paper.

## Abstract (precis)

**Summary:** Expression level modulation of the core paraspeckle protein SFPQ regulates sense and antisense RNA foci and dipeptide repeat protein accumulation in the *C9orf72* mutation; SFPQ could be a therapeutic target in *C9orf72* ALS and FTD.

## INTRODUCTION

Amyotrophic lateral sclerosis (ALS) and frontotemporal dementia (FTD) are fatal neurodegenerative disorders. As they share clinical, genetic and pathological features, ALS and FTD are often presented as two ends of a spectrum disorder (Abramzon et al., 2020; Gao et al., 2017). Mutations of over 50 genes involved in RNA metabolism, protein quality control and turnover have been implicated in ALS and FTD (Boylan, 2015; Mandrioli et al., 2020; Ramaswami et al., 2013). The most common genetic cause is a mutation in the *C9orf72* gene, which consists of expanded repeats of the hexanucleotide GGGGCC sequence in the non-coding region of the *C9orf72* gene. Up to 20–25 of these repeats are present in healthy individuals, whereas up to several thousand repeats occur in *C9orf72* ALS and FTD patients (DeJesus-Hernandez et al., 2011; Gao et al., 2017; Renton et al., 2011). These repeats form secondary structures, such as G-quadruplexes, hairpins and i-motifs, both at the DNA and RNA level (Božič et al., 2020; Haeusler et al., 2014; Kovanda et al., 2016; Šket et al., 2015). Three mechanisms of action have been proposed for the *C9orf72* mutation (Balendra and Isaacs, 2018). First, the mutation leads to haploinsufficiency of the C9ORF72 protein, presumably due to decreased levels of mRNA from the mutant allele (Balendra and Isaacs, 2018; Donnelly et al., 2013; Tran et al., 2015; Waite et al., 2014). Second, sense and antisense RNA transcripts from mostly nuclear RNA foci in *C9orf72* ALS and FTD (Balendra and Isaacs, 2018; DeJesus-Hernandez et al., 2011; Donnelly et al., 2013; Gendron et al., 2013; Lagier-Tourenne et al., 2013; Lee et al., 2013; Mizielinska et al., 2013; Vatovec et al., 2014; Zu et al., 2013). It has been proposed that these foci sequester RNA-binding proteins and alter their function, a process referred to as RNA toxicity (Gao et al., 2017; Haeusler et al., 2016; Lee et al., 2013; Todd and Paulson, 2010). Multiple studies have shown that various proteins bind to sense and antisense RNA from the *C9orf72* mutation (DeJesus-Hernandez et al., 2011; Donnelly et al., 2013; Gendron et al., 2013; Haeusler et al., 2014; Lee et al., 2013; Mizielinska et al., 2013; Mori et al., 2013a; Swinnen et al., 2018; Xu et al., 2013; Zhang et al., 2015; Zu et al., 2013). Third, expanded repeat sense and antisense RNA is translated to dipeptide repeat (DPR) proteins by repeat-associated non-AUG translation. Sense RNA translates to poly-glycine-alanine (pGA), poly-glycine-arginine (pGR) and poly-glycine-proline (pGP) DPRs, and antisense RNA translates to poly-proline-glycine (pPG), poly-proline-arginine (pPR) and poly-proline-alanine (pPA). DPRs form insoluble deposits in patient neurons and glia and exert toxic effects in disease models (Al-Sarraj et al., 2011; Ash et al., 2013; Balendra and Isaacs, 2018; Brasseur et al., 2020; Gao et al., 2017; Gendron et al., 2013; Haeusler et al., 2016; Lee et al., 2020; Mori et al., 2013b,c; Saberi et al., 2018; Suzuki et al., 2019; Zu et al., 2013). The above-mentioned mechanisms of action of the *C9orf72* mutation are predicted to act in synergy to provoke disease-relevant phenotypes, as opposed to only one of the described mechanisms playing the predominant role (Balendra and Isaacs, 2018; Haeusler et al., 2016).

The mutation in *C9orf72* and mutations in three other genes – *TDP-43* (TAR DNA-binding protein 43), *SOD1* (superoxide dismutase) and *FUS* (fused in sarcoma) – account for approximately two-thirds of familial ALS cases (Hardiman et al., 2017). TDP-43 and FUS are RNA-binding proteins that play a role in multiple RNA processing and metabolic pathways and form cytoplasmic inclusions and nuclear depletion in ALS and FTD (Gao et al., 2017; Kwiatkowski et al., 2009; Ling et al., 2013; Mitchell et al., 2013; Neumann et al., 2009; Rogelj et al., 2012; Sreedharan et al., 2008; Štalekar et al., 2015; Vance et al., 2009). Recent studies have indicated the involvement of another RNA-binding protein, splicing factor proline and glutamine rich (SFPQ), in ALS pathology (Ishigaki et al., 2017; Lee et al., 2015; Luisier et al., 2018). SFPQ exhibits intron retention and nuclear loss in ALS and aberrant localization to the cytoplasm and formation of insoluble structures in FTD (Lee et al., 2015; Luisier et al., 2018). These are well-known ALS and FTD pathologies previously observed for TDP-43 and FUS (Li et al., 2013). Mutations of these proteins have been linked to misregulation of gene expression in cell models and motor neurons in ALS and FTD (Lee et al., 2015; Li et al., 2013; Prpar Mihevc et al., 2016; Ramaswami et al., 2013). SFPQ is an abundant nuclear protein with various functions, including alternative splicing, DNA repair, transcriptional regulation and RNA processing and transport (Haeusler et al., 2016; Hirose et al., 2014; Lee et al., 2015; Shav-Tal and Zipori, 2002). SFPQ is one of the core proteins of paraspeckles – nuclear organelles formed by RNA–protein and protein–protein interactions on long non-coding RNA *NEAT1_2* scaffold (Bond and Fox, 2009; Clemson et al., 2009; Fox et al., 2018; Mao et al., 2011; West et al., 2016). An increase in *NEAT1_2* levels leads to the sequestration of more paraspeckle proteins, which in the case of SFPQ reduces its relative levels in the cytoplasm and changes the expression of its target genes (Fox et al., 2018). *NEAT1_2* up-regulation and increased paraspeckle formation occur in the early phase of ALS (Nishimoto et al., 2013; Shelkovnikova et al., 2018). Mutations in other paraspeckle proteins also lead to their aggregation or mislocalization to the cytoplasm in ALS, indicating a strong involvement of these structures in the underlying mechanisms of ALS (Fox et al., 2018; Li et al., 2013; Modic et al., 2019; Naganuma et al., 2012; Nishimura et al., 2010; Shelkovnikova et al., 2014).

Expanded RNA repeats from the *C9orf72* gene have been shown to interact with SFPQ across studies (Haeusler et al., 2016), and we have recently shown that sense RNA foci form paraspeckle-like nuclear bodies by sequestering paraspeckle proteins (Bajc Česnik et al., 2019). In the current study, we show that SFPQ regulates the formation of both sense and antisense RNA foci as well as the accumulation of all five hexanucleotide repeat-encoded DPRs. Therefore, it presents a potential therapeutic target for expression level modulation in *C9orf72* ALS and FTD.

## RESULTS

### SFPQ does not bind antisense RNA *in vitro*

**Fig. 1.**
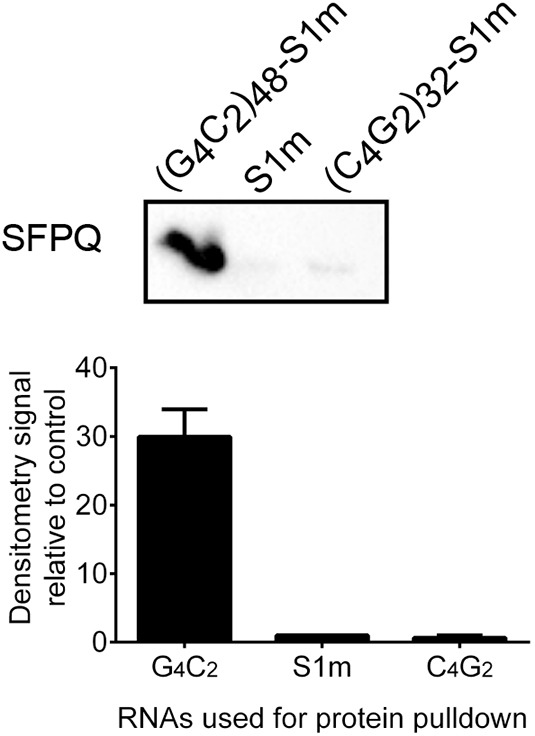
**SFPQ binds to sense but not antisense *C9orf72* repeat RNA.** The RNA constructs (G_4_C_2_)_48_–S1m, (C_4_G_2_)_32_–S1m and S1m were used. The immunoblot confirms SFPQ binding to G_4_C_2_ RNA repeats, as the signal is 29.94±4.04 relative to the control, whereas SFPQ is not bound by C_4_G_2_, as the signal is 0.66±0.33 relative to the control S1m tag. The experiment was replicated three times in the laboratory. Error bars denote s.d.

First, we investigated whether SFPQ interacts with antisense RNA *in vitro*, as is the case for sense RNA (Bajc Česnik et al., 2019), by performing a RNA pull-down assay on mouse brain lysates. Sense and antisense RNA repeat constructs both containing the S1m tag, and an RNA construct of only the S1m tag were used for this purpose. We used constructs with long (G_4_C_2_)_48_ and (C_4_G_2_)_32_ RNA repeats for the RNA pull-down. These were the longest constructs containing S1m aptamer that were stable for cloning. SFPQ did not bind to the (C_4_G_2_)_32_ RNA repeats, as the signal for SFPQ was 0.66±0.33 (mean±s.d.) relative to control S1m construct, whereas SFPQ did bind to the (G_4_C_2_)_48_ RNA repeats, as the signal increased to 29.94±4.04 of the control (Fig. 1; Fig. S1).

### SFPQ knockdown reduces RNA foci number and DPR expression in HEK cells

**Fig. 2.**
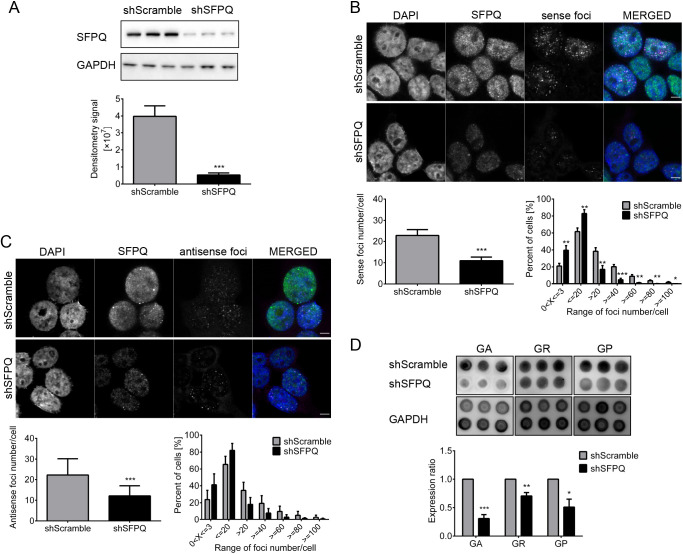
**SFPQ knockdown in HEK293T cells reduces the number of sense and antisense foci and dipeptide repeat protein (DPR) expression.** HEK293T cells expressing (G_4_C_2_)_72_ or (C_4_G_2_)_32_ repeats treated with either shScramble (control) or shSFPQ (SFPQ KD) are presented. (A) shSFPQ reduces the expression level of SFPQ to 0.13±0.02 relative to that in shScramble-treated cells. (B) The number of sense foci is 10.94±1.71 in SFPQ KD and 22.78±2.74 in control cells. A higher percentage of SFPQ KD cells have <20 sense foci per cell, whereas a higher percentage of control cells have >20 foci per cell. *n* for control cells=3390, *n* for KD cells=3821. (C) The number of antisense foci is 12.12±4.88 in SFPQ KD and 22.18±8.01 in the control cells. A higher percentage of SFPQ KD cells have <20 antisense foci per cell, whereas a higher percentage of control cells have >20 foci per cell. *n* for control cells=3680, *n* for KD cells=4215. (D) The ratios of DPR expression levels in SFPQ KD versus control cells are 0.31±0.07 (pGA), 0.71±0.06 (pGR) and 0.51±0.14 (pGP). Scale bars: 5 µm. The experiments are performed for three biological (three different passages of HEK293T cells) and at least two technical repeats (two independent experiments performed on each cell line). Error bars denote s.d. Statistical significance is calculated with the two-tailed *t*-test and is labeled as: *P<0.05, **P<0.01, ***P<0.001.

To evaluate the impact of SFPQ knockdown on the number of sense and antisense foci as well as levels of DPRs, we used lentiviral particles harboring shSFPQ for SFPQ knockdown, and shScramble was used as a control. Cells were transfected with plasmids harboring (G_4_C_2_)_72_ or (C_4_G_2_)_32_ repeats. The plasmid harboring (G_4_C_2_)_72_ was used for its ability to be translated to sense-derived DPRs. Expression level of SFPQ in SFPQ KD cells was reduced to 0.13±0.03 (mean±s.d.) of the controls (Fig. 2A; Fig. S2). There was a significant reduction in both sense and antisense foci number: 10.94±1.71 (mean±s.d.) sense foci per cell in SFPQ KD cells compared with 22.78±2.74 in controls, and 12.12±4.88 antisense foci per cell in SFPQ KD cells compared with 22.18±8.01 in controls (Fig. 2B,C). Therefore, the foci number was reduced by approximately half. There was also a significant impact on the range of sense foci per cell, as a higher percentage of SFPQ KD cells had <20 foci per cell, whereas a higher percentage of control cells had >20 foci per cell (Fig. 2B). The same trend was observed for antisense foci; however, there was no significance due to the high variability of the biological replicates (Fig. 2C). Furthermore, the knockdown of SFPQ reduced the synthesis of DPR proteins (Fig. 2D). Compared with the control, the expression levels were 0.31±0.07 (mean±s.d.; pGA), 0.71±0.06 (pGR) and 0.51±0.14 (pGP) (Fig. 2D).

### SFPQ knockdown reduces RNA foci number and DPR expression in *C9orf72* mutation-positive cells

**Fig. 3.**
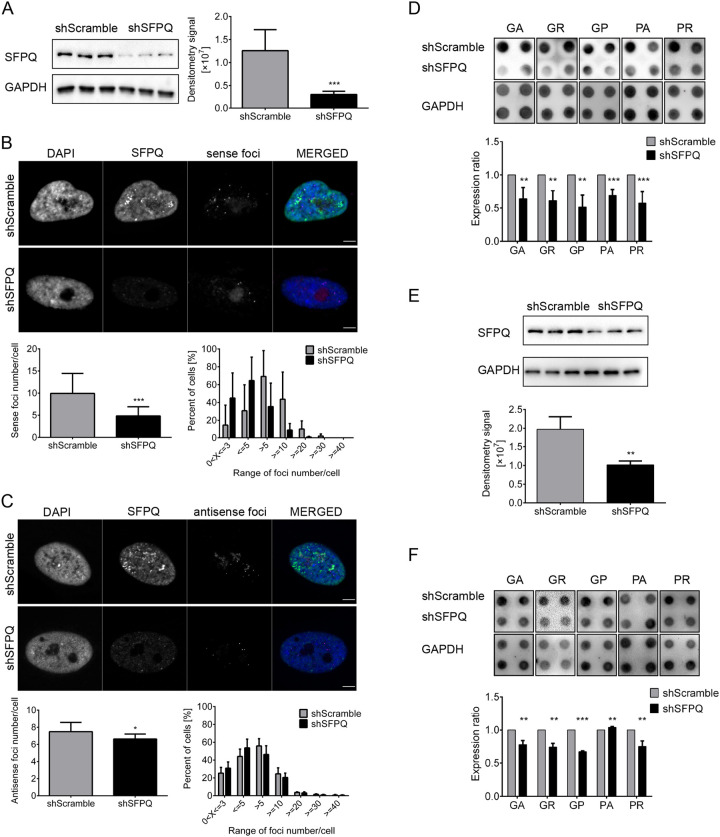
**SFPQ knockdown in *C9orf72* mutation-positive patient-derived cells reduces the number of sense and antisense foci and DPR expression.** *C9orf72* mutation-positive patient-derived fibroblasts and lymphoblasts treated with either shScramble (control) or shSFPQ (SFPQ KD) are presented. (A) The expression level of SFPQ in SFPQ KD fibroblasts is reduced to 0.27±0.12 relative to controls. (B) SFPQ KD reduces the number of sense foci per cell from 9.93±4.51 in control to 4.48±2.06 in SFPQ KD fibroblasts. A higher percentage of SFPQ KD cells have <5 sense foci per cell, whereas a higher percentage of control cells have >5 foci per cell. *n* for control cells=299, *n* for KD cells=199. (C) The number of antisense foci/cell is reduced from 7.49±1.08 in control to 6.63±0.58 in SFPQ KD fibroblasts. Higher percentage of SFPQ KD cells has <5 foci per cell, whereas a higher percentage of control cells have >5 foci per cell. *n* for control cells=735, *n* for KD cells=675. (D) The ratios of DPR expression levels in SFPQ KD versus control fibroblasts are 0.64±0.17 (pGA), 0.61±0.15 (pGR), 0.51±0.18 (pGP), 0.69±0.09 (pPA) and 0.58±0.17 (pPR). (E) SFPQ expression levels are reduced to 0.52±0.08 in SFPQ KD relative to control lymphoblasts. (F) The ratios of DPR expression levels in SFPQ KD versus control lymphoblasts are 0.78±0.06 (pGA), 0.74±0.06 (pGR), 0.67±0.01 (pGP), 1.04±0.01 (pPA) and 0.75±0.09 (pPR). Scale bars: 5 µm. The experiments are performed for three biological (three different fibroblast cell lines and three different lymphoblast cell lines) and at least two technical repeats (two independent experiments performed on each cell line) Error bars denote s.d. Statistical significance is calculated with the two-tailed *t*-test and is labeled as: *P<0.05, **P<0.01, ***P<0.001.

We have previously shown that SFPQ knockdown reduces the number of sense foci in fibroblasts (Bajc Česnik et al., 2019). Here, we have confirmed our previous observation with a more detailed analysis and have expanded our observations to antisense foci. We used lentiviral particles for SFPQ knockdown in *C9orf72* mutation-positive patient-derived cells; shScramble was used as a control. Expression level of SFPQ was reduced to 0.27±0.12 (mean±s.d.) in SFPQ KD fibroblasts compared with control (Fig. 3A; Fig. S3A); and to 0.52±0.08 in SFPQ KD lymphoblasts compared with control (Fig. 3E; Fig. S3B). Reduced expression of SFPQ caused reduction of sense foci number from 9.93±4.51 (mean±s.d.) in control to 4.48±2.06 in SFPQ KD fibroblasts (Fig. 3B). High standard deviations were a consequence of different overall foci numbers in multiple *C9orf72* mutation-positive patient-derived fibroblast lines used. Nevertheless, there was a significant sense foci reduction relative to control in all lines used, as sense foci number was reduced by half (ratio 0.5±0.08) in SFPQ KD fibroblasts compared with control (Fig. 3B). Reduced expression of SFPQ caused a smaller, but significant reduction of average antisense foci number per cell. The number of antisense foci was reduced from 7.49±1.08 in control to 6.63±0.58 foci per cell in SFPQ KD fibroblasts (Fig. 3C). SFPQ KD led to changes in the number of both sense and antisense foci in fibroblasts. There was a higher percentage of SFPQ KD fibroblasts harboring <5 foci per cell, and a higher percentage of control fibroblasts harboring >5 foci per cell. However, the difference was more distinct for sense foci (Fig. 3B,C). We also observed reduced expression of DPRs in SFPQ KD fibroblasts (Fig. 3D). Compared with controls, the expression levels were 0.64±0.17 (mean±s.d.; pGA), 0.61±0.15 (pGR), 0.51±0.18 (pGP), 0.69±0.09 (pPA) and 0.58±0.17 (pPR) (Fig. 3D). Furthermore, the expression levels of four DPRs were significantly reduced in SFPQ KD lymphoblasts compared with controls; the expression levels were 0.78±0.06 (pGA), 0.74±0.06 (pGR), 0.67±0.01 (pGP), 1.04±0.01 (pPA) and 0.75±0.09 (pPR) (Fig. 3F).

### SFPQ over-expression increases RNA foci number and DPR expression in HEK293T cells

**Fig. 4.**
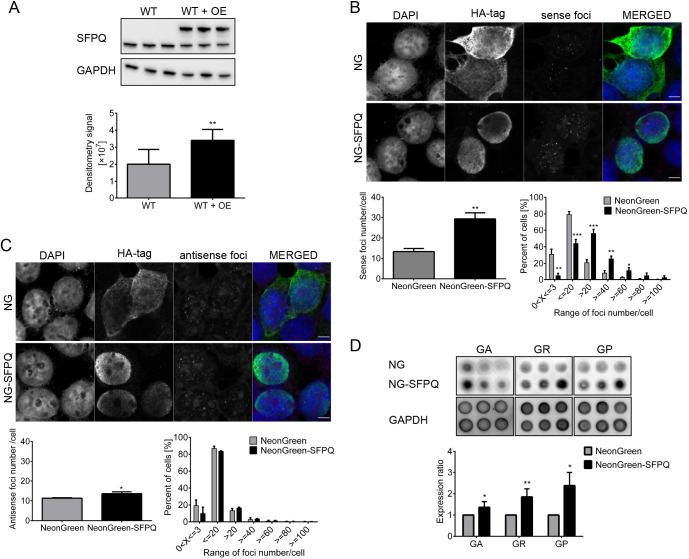
**SFPQ over-expression (OE) in HEK293T cells increases the number of sense and antisense foci and DPR expression.** HEK293T cells expressing (G_4_C_2_)_72_ or (C_4_G_2_)_32_ repeats transfected with either NeonGreen (control) or NeonGreen-SFPQ (SFPQ OE) are presented. (A) SFPQ OE cells exhibit 1.87±0.51 times higher SFPQ expression relative to control cells. (B) SFPQ over-expression leads to the formation of 29.37±2.97 sense foci per cell, whereas 13.35±1.50 sense foci per cell were formed in control cells. A higher percentage of SFPQ OE cells have >20 sense foci per cell, whereas a higher percentage of control cells have <20 foci per cell. *n* for control cells=1300, *n* for OE cells=920. (C) SFPQ OE leads to the formation of 13.58±0.94 antisense foci per cell compared with 11.32±0.22 antisense foci per cell in control cells. Conversely, the distribution of antisense foci per cell does not significantly differ between SFPQ OE and control cells. *n* for control cells=1415, *n* for OE cells=1012. (D) The ratios of DPR expression levels in SFPQ OE versus control cells are 1.36±0.27 (pGA), 1.85±0.38 (pGR) and 2.38±0.62 (pGP). NG, NeonGreen; NG-SFPQ, NeonGreen-SFPQ. Scale bars: 5 µm. The experiments are performed for three biological (three different passages of HEK293T cells) and at least two technical repeats (two independent experiments performed on each cell line). Error bars denote s.d. Statistical significance is calculated with the two-tailed *t*-test and is labeled as: *P<0.05, **P<0.01, ***P<0.001.

To further examine the impact of SFPQ expression levels on RNA foci formation and DPR expression, we over-expressed SFPQ in HEK293T cells. Cells were co-transfected with plasmids harboring (G_4_C_2_)_72_ or (C_4_G_2_)_32_ repeats and plasmids harboring NeonGreen-SFPQ or NeonGreen only, hereafter referred to as SFPQ OE and control, respectively. We successfully over-expressed SFPQ, which was 1.87±0.51 times more expressed in SFPQ OE relative to control cells (Fig. 4A; Fig. S4). SFPQ OE led to significantly increased numbers of sense and antisense foci relative to control cells; however, the increase was smaller for antisense than sense foci. The number of sense foci per cell was 29.37±2.97 (mean±s.d.) in SFPQ OE and 13.35±1.50 in control cells (Fig. 4B). The number of antisense foci per cell was 13.58±0.94 (mean±s.d.) in SFPQ OE and 11.32±0.22 in control cells (Fig. 4C). Therefore, SFPQ overexpression led to the formation of 2.24±0.50 times more sense foci per cell and 1.2±0.1 times more antisense foci per cell relative to control cells. Additionally, there was a significantly higher percentage of SFPQ OE cells with >20 sense foci per cell compared with control cells, whereas there was no significant change in antisense foci (Fig. 4B,C). Furthermore, we detected increased DPR protein synthesis. Compared with controls, the expression levels were 1.36±0.27 (mean±s.d.; pGA), 1.85±0.38 (pGR) and 2.38±0.62 (pGP) (Fig. 4D).

## DISCUSSION

This study expands our knowledge on the role of the key paraspeckle protein SFPQ in the *C9orf72* expanded repeat mutation. In addition to our previous findings that have shown that SFPQ interacts with sense RNA foci during the formation of paraspeckle-like bodies (Bajc Česnik et al., 2019), we have now shown for the first time that SFPQ affects the number of not only sense but also antisense RNA foci, as well as the production of DPRs in transfected HEK cells and *C9orf72* mutation-positive patient-derived cells. Reduced levels of SFPQ reduce the number of sense and antisense foci and DPR accumulation, whereas SFPQ over-expression increases the formation of sense and antisense foci and DPRs.

The correlation between sense RNA foci formation and SFPQ levels could be explained by their direct interaction, which has been published previously (Bajc Česnik et al., 2019). SFPQ and sense foci interact within paraspeckle-like bodies, in which sense foci sequester SFPQ and act as scaffold RNA instead of *NEAT1_2* (Bajc Česnik et al., 2019). Changes in SFPQ levels could influence the formation of these bodies and, thus, the number of sense RNA foci per cell.

Conversely to sense RNA, we did not detect *in vitro* interaction between antisense RNA and SFPQ by RNA pull-down assay. This could be the consequence of secondary structures formed by sense and antisense RNA, which have been proposed to enable their interaction with various RNA binding proteins (McEachin et al., 2020; Vatovec et al., 2014). Sense RNA forms mostly G quadruplexes and hairpins, whereas antisense RNA forms i-motifs along with C-rich hairpins (Božič et al., 2020; Haeusler et al., 2014; Kovanda et al., 2016; Šket et al., 2015). In fact, SFPQ has been previously shown to preferentially bind to G quadruplex formations (Simko et al., 2020). Moreover, SFPQ could impact the stability of RNA foci as it has been suggested that also stability of *NEAT1* depends on core paraspeckle proteins binding along its structure (Fox et al., 2018; Sasaki et al., 2009).

Nevertheless, we observed that SFPQ expression level affected the number of antisense foci in cells as well, although to a lesser extent. Due to no direct interaction observed between antisense RNA foci and SFPQ, we conclude that additional or different pathways are involved in the impact of SFPQ levels on the number of antisense foci. One possibility is on account of SFPQ acting as a transcriptional regulator of repeats. Previous findings have demonstrated that SFPQ acts as a transcriptional regulator, exemplified by *NEAT1_2* sequestering SFPQ from promoter regions, thereby influencing the transcription of SFPQ-dependent genes (Hirose et al., 2014; Imamura et al., 2014; Lee et al., 2015; Song et al., 2005). Moreover, SFPQ enables the transcription of genes with long or structurally complex intron regions (Takeuchi et al., 2018), making it a strong candidate for enabling transcription of hexanucleotide repeat expansions to RNA in ALS and FTD. Transcriptional regulation in combination with paraspeckle-like body formation in the case of sense RNA could explain our results that revealed a larger effect of SFPQ on the formation of sense foci in comparison with antisense foci.

Furthermore, expression levels of SFPQ influenced the expression of DPRs. Other proteins involved in transcription have been previously associated with the number of RNA foci and production of DPRs (Kramer et al., 2016; Mori et al., 2016; Yuva-Aydemir et al., 2019). These findings suggest that SFPQ affects the availability of both sense and antisense RNA for translation. On the one hand, this could be the consequence of reduced G_4_C_2_ sequence transcription as discussed before. On the other hand, SFPQ could impact the transport of repeat RNAs or impact their translation directly. SFPQ has been previously shown to play a role in the RNA transport granules regulating mRNA translation (Cosker et al., 2016; Kanai et al., 2004). It has also been reported that SFPQ is recruited into stress granules, which are involved in regulation of protein translation (Younas et al., 2020).

Our findings raise interesting questions regarding the role of SFPQ in the *C9orf72* mutation. For instance, it is possible that sense RNA foci function similarly to *NEAT1_2* and, by sequestering SFPQ and other proteins, foci may regulate the transcription of different genes and their own, acting as a negative loop. It has been previously suggested that RNA foci act as a toxic RNA sink, providing neuroprotection by reducing DPR production, and DPRs have been associated with toxicity in several studies (Balendra and Isaacs, 2018; Brasseur et al., 2020; Gendron et al., 2013; Kino et al., 2015; Moens et al., 2018). However, the mechanism of this action has not yet been shown. In addition, pPR has been shown to up-regulate *NEAT1_2*, forming a possible negative feedback loop for DPR production via *NEAT1_2* sequestration of SFPQ (Suzuki et al., 2019).

Our results suggest that modulating the expression levels of SFPQ is a potential therapeutic target for *C9orf72* ALS and FTD patients. A previous study has shown that Spt4 has a similar impact on sense and antisense RNA levels and expression of DPRs in patient cells (Kramer et al., 2016). The advantage of regulating transcription of the *C9orf72* mutation through modulation of transcription factors is simultaneous impact on both sense and antisense RNA and corresponding DPRs compared with sequence-specific impact of antisense oligonucleotides (ASOs), which are so far the major therapeutic approach for reduction of RNA foci (Kramer et al., 2016; Riboldi et al., 2014). Altogether, our findings contribute to understanding the role of SFPQ in ALS and FTD, and provide a potential therapeutic perspective for SFPQ in *C9orf72* ALS and FTD.

## MATERIALS AND METHODS

### Plasmids

The construct pcDNA3.2/GW/D-TOPO containing (G_4_C_2_)_72_ and construct pcDNA3.1 containing (G_4_C_2_)_48_ with S1 aptamer on one end have been described previously (Lee et al., 2013). We prepared construct pcDNA3.1 containing (C_4_G_2_)_32_ and (G_4_C_2_)_48_ with S1m aptamer from previously described plasmids. The constructs pcDNA5/FRT/TO-NeonGreen-SFPQ-3xHA and pcDNA5/FRT/TO-NeonGreen-3xHA were prepared from plasmid pL40C_PGKintron_Cas9_Green (a gift from Beat Bornhauser, Addgene plasmid no. 134966; RRID: Addgene_134966) (Huang et al., 2019) and plasmid Myc-PSF-WT (a gift from Benjamin Blencowe, Addgene plasmid no. 35183; RRID: Addgene_35183) (Rosonina et al., 2005). The plasmids pMD2.G (deposited by Didier Trono, Addgene plasmid no. 12259; RRID: Addgene_12259) and psPAX2 (deposited by Didier Trono; Addgene plasmid no. 12260; RRID: Addgene_12260) were kindly provided by Dr Don W. Cleveland (Ludwig Institute for Cancer Research, La Jolla, CA, USA). Scramble shRNA (short hairpin RNA) was a gift from David Sabatini (Addgene plasmid no. 1864; RRID: Addgene_1864) (Sarbassov et al., 2005). SFPQ shRNA was NM_005066.x977s1c1 (Sigma-Aldrich, St Louis, MO, USA).

### Antibodies

The following commercial antibodies were used: SFPQ-specific mouse monoclonal antibody [sc-374502, Santa Cruz Biotechnologies, Dallas, TX, USA; western blotting (WB) 1:500, immunocytochemistry (ICC) 1:100], anti-HA tag rabbit polyclonal antibody (NB600-363, Novus Biologicals, Centennial, CO, USA; WB 1:1000, ICC 1:500), pGA repeat rabbit polyclonal antibody (24492-1-AP, Proteintech, Rosemont, IL, USA), pGR repeat rabbit polyclonal antibody (23978-1-AP, Proteintech), pGP repeat rabbit polyclonal antibody (24494-1-AP, Proteintech), pAP repeat rabbit polyclonal antibody (24493-1-AP, Proteintech) and pPR repeat rabbit polyclonal antibody (23979-1-AP, Proteintech). All DPR antibodies were used at a 1:1000 dilution for dot blots, including GAPDH rabbit polyclonal antibody (10494-1-AP, Proteintech), GAPDH mouse monoclonal antibody (60004-1-Ig, Proteintech), anti-mouse IgG (H+L), F(ab′)2 Fragment (Alexa Fluor 488 Conjugate) (4408, Cell Signaling Technology, Danvers, MA, USA; dilution 1:1000), StarBright Blue 520 Fluorescent Secondary Antibodies (nos. 12005866 and 12005869, Bio-Rad, Hercules, CA, USA; dilution 1:5000), StarBright Blue 700 Fluorescent Secondary Antibodies (nos. 12004158 and 12004161, Bio-Rad; dilution 1:5000) and Peroxidase AffiniPure Goat Anti-Rabbit IgG (H+L) (111-035-045, Jackson ImmunoResearch, West Grove, PA, USA; dilution 1:5000).

### Cell culture, transfection and lentiviral production

HEK293T lentivirus production cells (ATTC, Manassas, VA, USA; a kind gift from Dr Don W. Cleveland) were maintained in high-glucose Dulbecco's modified Eagle's medium (DMEM, GlutaMax; Gibco, Thermo Fisher Scientific, Waltham, MA, USA) supplemented with 10% fetal bovine serum (Gibco, Thermo Fisher Scientific) and 100 U ml^−1^ penicillin–streptomycin (Gibco, Thermo Fisher Scientific). *C9orf72* mutation-positive fibroblasts were a kind gift from Dr Don W. Cleveland (Lagier-Tourenne et al., 2013). Fibroblasts were maintained in DMEM supplemented with 20% fetal bovine serum and 100 U ml^−1^ penicillin–streptomycin. *C9orf72* mutation-positive lymphoblasts were a kind gift from Guy Rouleau (McGill University, Department of Neurology & Neurosurgery, Montreal Neurological Institute, Montreal, Canada). Lymphoblasts were maintained in RPMI 1640 (Gibco, Thermo Fisher Scientific) supplemented with 15% fetal bovine serum and 100 U ml^−1^ penicillin–streptomycin. All cell lines were tested and confirmed to be mycoplasma-free. HEK293T cells were seeded onto poly-l-lysine-coated (Sigma-Aldrich) glass coverslips and transfected with Lipofectamine 2000 (Life Technologies, Carlsbad, CA, USA) for RNA fluorescence *in situ* hybridization with immunocytochemistry (RNA-FISH/ICC) experiments. For SFPQ knockdown experiments, HEK293T lentivirus production cells were seeded to reach 70–90% confluence; after 24 h, they were co-transfected with the plasmids for lentiviral production pMD2.G, psPAX2 and pLKO.2 shSFPQ or pLKO.1 shScramble at 1:2:3 ratios with PolyJet transfection reagent (SL100688, SignaGen Laboratories, Frederick, MD, USA) according to the manufacturer's instructions. Medium was replaced 6 h post-transfection with target cell growth medium, cells were grown for another 48 h, when the supernatant was collected, filtered through a 0.45 µm cellulose acetate membrane, diluted 3:1 with fresh target cell medium and added to the target cells, seeded a day before. The medium was replaced with fresh medium after 24 h. The cells were grown for another 65 h and then collected for western blot or RNA-FISH/ICC.

### RNA pull-down assay

RNA pull-down was performed on mouse brain lysates as described previously (Bajc Česnik et al., 2019; Lee et al., 2013). For each RNA pull-down experiment, 400 mg of mouse brain tissue was used. Mouse brain tissue nuclear extracts were prepared as described by Lee et al. (2013). The plasmids pcDNA3.1(C_4_G_2_)_32_–S1m, pcDNA3.1(G_4_C_2_)_48_–S1m and pcDNA3.1–S1m were linearized at the restriction site on the 3′-end of the S1m aptamer. The constructs contained the T7 promotor at the 5′-end and were transcribed to RNA with the TranscriptAid T7 High Yield Transcription Kit (Fermentas, Waltham, MA, USA). Single-strand binding protein (Sigma-Aldrich) was added at a concentration of 7.5 μg per 1 μg of DNA for the transcription of GC-rich hexanucleotide repeats; reactions were performed for 6 h at 42°C for (G_4_C_2_)_48_–S1m and at 37°C for (C_4_G_2_)_32_–S1m and S1m. RNA constructs were diluted in RNA-binding buffer [50 mM Hepes (pH 7.4), 100 mM KCl, 10 mM MgCl_2_, 0.5% IGEPAL CA-630] and incubated with streptavidin magnetic beads (Promega, Madison, WI, USA) for 30 min at room temperature (RT). Following RNA binding, the beads were washed with RNA-binding buffer and incubated with mouse nuclear brain extract for 4 h at 4°C. RiboLock RNase inhibitor (Fermentas) (20 U) and 50 μg yeast tRNA (Sigma-Aldrich) were added to extract. The beads were then washed with RNA-binding buffer, and the bound proteins were eluted by incubating the beads with 3 U RNase I (EN0601, Thermo Fisher Scientific) per reaction for 10 min at 37°C. Eluates were then prepared for western blot by adding loading buffer. Mouse brains were obtained under approval of the Veterinary Administration of the Ministry of Agriculture and the Environment, Slovenia.

### Immunoblotting

Protein samples were prepared in 2×SDS loading buffer and 200 mM dithiothreitol, incubated at 95°C for 5 min, and separated on 12% SDS precast gels (Invitrogen, Carlsbad, CA, USA) run at 125 V. Wet transfer onto nitrocellulose membrane (GE Healthcare, Chicago, IL, USA) was performed at 200 mA for 90 min. Dot blots were performed by adding protein lysates dropwise onto the membrane, followed by 15 min of drying. The membranes were blocked in 5% skim milk in 0.05% Tween-20 for 1 h at RT and then incubated with the primary antibodies in blocking buffer overnight at 4°C with gentle rocking. The membranes were washed 3×10 min with washing buffer (0.05% Tween-20) and incubated for 1 h with the secondary antibodies in blocking buffer. The membranes were then washed 3×10 min with washing buffer and incubated with Clarity Max Western ECL Substrate (Bio-Rad). Images were acquired by the GelDoc System (Bio-Rad), and ImageLab software (Bio-Rad) was used for densitometric analysis. For each experiment, three biological repeats and at least two technical repeats were performed.

### RNA-FISH/ICC

Locked nucleic acid (LNA) FISH probes were purchased from Exiqon: 5TYE563, 5′-GGGGCCGGGGCCGGGG-3′; 5TYE563, 5′-CCCCGGCCCCGGCCCC-3′. Cells were fixed in 4% paraformaldehyde in phosphate-buffered saline (PBS) for 15 min and permeabilized with 0.1% Triton X-100 in PBS for 5 min. The coverslips were incubated in pre-hybridization solution [40% formamide, 2× saline sodium citrate (SSC)] for 15 min, followed by overnight hybridization with 2 µM of G_4_C_2_/C_4_G_2_ probe diluted and heated (95°C, 5 min) in hybridization solution [2×SSC, 100 μg ml^−1^ tRNA (R8508, Sigma), 10% dextran sulfate, 25% formamide] overnight at 60°C. Stringency washes were performed the following day: 1×5 min in 2×SSC, 0.1% Tween-20 at RT and 3×10 min in 0.1×SSC at 60°C. Cells were stained with DAPI at RT for 10 min, washed for 10 min with PBS, and mounted with ProLong Gold antifade reagent (Thermo Fisher Scientific). For ICC, cells were briefly washed with 2×SSC after stringency washes and blocked in 10% goat serum (Sigma-Aldrich) in PBS for 30 min at RT. Primary and secondary antibodies were incubated in 5% goat serum (Sigma-Aldrich) in PBS for 1 h at RT. After incubation with primary antibodies, cells were washed 3×5 min with PBS. Secondary anti-rabbit Alexa Fluor 488 antibodies were used. Images were acquired with a Zeiss LSM 710 inverted confocal laser scanning microscope with a Plan-Apochromat 63× and 1.4 NA M27 oil immersion objective using immersion oil (Carl Zeiss) and Leica TCS SP8 Plan apo 63× and 1.4 NA CS2 oil immersion objective. DAPI, Alexa Fluor 488 and 5TYE563 were excited at 405, 488 and 543 nm, respectively. The zoom factor was set to 1–4×, and X- and Y-scanning sizes were each 1024 pixels. All images were acquired as z-stacks, and the z-scanning size was 0.979–2 μm.

### Quantification and statistical analysis

Densitometric values of protein bands in immunoblotting were normalized to GAPDH using ImageLab 5.1 software (Bio-Rad). Relative protein expression levels in SFPQ knock-down (SFPQ shRNA) conditions were calculated relative to shRNA scramble controls. At least three biological repeats consisting of three different cell lines (three different passages of HEK293T cells, three fibroblast lines or three lymphoblasts lines) were used. Two technical repeats, which present two independent experiments performed on each cell line used, were performed for each experiment. For RNA-FISH fluorescence quantification of the number of RNA foci, the ImageJ functions multiple points and find maxima were used on each slice of the z-stacks, and the counted foci were summed for each cell through the stacks. Cell counts were performed for three biological and at least two technical repeats; at least 100 cells were counted per experiment. Statistical significance of the differential expression of dipeptide repeat proteins according to dot blot and RNA foci number according to RNA-FISH was determined with unpaired, two-tailed Student's *t*-test in Microsoft Excel 2010. *P*<0.05 was considered significant. All values and graphs present mean values±s.d., and statistical significance is indicated as follows: *P<0.05, **P<0.01, ***P<0.001.

## Supplementary Material

Reviewer comments
