# Supplementary material for: SFPQ regulates the accumulation of RNA foci and dipeptide repeat proteins from the expanded repeat mutation in C9orf72
Source: J Cell Sci. 2021 Feb 19;134(4):jcs256602. doi: 10.1242/jcs.256602 (PMC7904093; doi:10.1242/jcs.256602)
Supplement: Reviewer comments [file joces-134-256602_review_history.pdf]

SFPQ regulates the accumulation of RNA foci and dipeptide repeat proteins from the expanded repeat mutation in *C9orf72*

Mirjana Malnar and Boris Rogelj
DOI: 10.1242/jcs.256602

Editor: Maria Carmo-Fonseca

Review timeline

Original submission:	2 November 2020
Editorial decision:	7 December 2020
First revision received:	10 December 2020
Accepted:	8 January 2021

Original submission

First decision letter

MS ID#: JOCES/2020/256602

MS TITLE: SFPQ regulates the accumulation of RNA foci and dipeptide repeat proteins from the expanded repeat mutation in C9orf72

AUTHORS: Mirjana Malnar and Boris Rogelj
ARTICLE TYPE: Research Article

We have now reached a decision on the above manuscript.

To see the reviewers' reports and a copy of this decision letter, please go to: https://submit-jcs.biologists.org and click on the 'Manuscripts with Decisions' queue in the Author Area. (Corresponding author only has access to reviews.)

I apologize for the delay in handling your manuscript. I have been waiting for the comments of the third assigned reviewer, but since we received no response, I have made a decision based on the two available reports. I have also carefully read the paper and in principle, I agree with reviewer #2 that further experiments are needed to strengthen the main conclusions of your study. If you think that you can deal satisfactorily with the criticisms raised by both reviewers, I would be pleased to see a revised manuscript. We would then return it to the reviewers.

We are aware that you may be experiencing disruption to the normal running of your lab that makes experimental revisions challenging. If it would be helpful, we encourage you to contact us to discuss your revision in greater detail. Please send us a point-by-point response indicating where you are able to address concerns raised (either experimentally or by changes to the text) and where you will not be able to do so within the normal timeframe of a revision. We will then provide further guidance. Please also note that we are happy to extend revision timeframes as necessary.

Please ensure that you clearly highlight all changes made in the revised manuscript. Please avoid using 'Tracked changes' in Word files as these are lost in PDF conversion.

I should be grateful if you would also provide a point-by-point response detailing how you have dealt with the points raised by the reviewers in the 'Response to Reviewers' box. Please attend to

© 2021. Published by The Company of Biologists under the terms of the Creative Commons Attribution License (https://creativecommons.org/licenses/by/4.0/).

all of the reviewers' comments. If you do not agree with any of their criticisms or suggestions please explain clearly why this is so.

Reviewer 1

Comments for the author

This is an interesting study by Malnar and Rogelj, which examines the role of SFPQ in regulating RNA foci and dipeptide repeat proteins (DPRs) from C9orf72 mutations. The main conclusions are 1) SFPQ overexpression results in increased RNA foci and DPRs in HEK293s; 2) SFPQ KD results in reduced RNA foci and DPRs in HEK293s, patient-derived C9orf72 mutant fibroblasts and lymphoblasts. The authors therefore very reasonably conclude that SFPQ has a role in regulating c9 mutation. Overall, this study is well conceived and clearly articulated. Moreover, it is important for the field and I am in favour of publication subject to relatively minor revisions / clarifications:
1) For the RIP experiments, it should be noted much SFPQ protein in each pull down. Also G4C2-48 plasmid used, then G4C2-72 later on – could the change be justified in the text please.
2) Regarding SFPQ KD in HEKs, could the KD data be shown so that the efficiency of KD can be visualised.
3) The authors show antisense foci reduction, but no SFPQ association with antisense foci on RIP – It is of course plausible that the role of SFPQ in foci production doesn’t necessitate direct interaction, but this should be discussed.
4) Could the authors clarify whether there was a decrease in PA or PR DPRs 5) In the SFPQ KD in C9 mutant cells, SFPQ appears to form nuclear puncta, which possibly colocalise with antisense RNA foci. However, how can this be reconciled with no RIP association?
6) In the Discussion, the following points should be considered:
- What role does sequestered SFPQ have in the stability of the RNA foci?
- Address lack of RIP interaction between SFPQ and anti-sense foci. Does this argue for different pathways of regulation of SFPQ upon anti or sense RNA foci.
- Is the relationship between SFPQ and DPRs downstream to the interaction of the protein with the RNAs?
- Do these data suggest a protective function as sequestered SFPQ would be unavailable to promote transcription of the c9 repeat RNAs and thereby DPR translation? Worth discussing further

Overall these experiments constitute a valid and robust approach to address role of SFPQ in levels of C9 associated species. The work is sound from an experimental perspective (power and appropriate methods used, over reliance on any one cell line is avoided through the use of fibroblasts, lymphoid cells and 293s).
Cumulatively this study strengthens the cause for SFPQ protein as a player in the most common ALS type (C9orf72), and whilst future studies would include further interrogation of the function SFPQ plays in production of these RNAs, and how it’s role differs between the sense and antisense species production, the current study adds important knowledge to the field.
Minor points
- The clockwise figure presentation is non-intuitive to follow.
- The authors should clarify which RNA repeat did and did not bind to SFPQ in the text on line 105.
- Please can the author explain why they have used a 48 repeat construct to look at SPFQ interaction in Figure 1 but then followed with a 72 repeat construct throughout the rest of the paper.
- The foci shown in figure 3C are difficult to see.
- Please can the author clarify what they mean by ‘biological repeats’ and ‘technical repeats’ and thus clarifying how many times the experiment was independently conducted.

Reviewer 2

Advance summary and potential significance to field

The relevance of SFPQ expression and intracellular localization of C9orf72 is interesting issue to understand the pathological mechanism of ALS/FTD. In this manuscript, the authors showed that expression level of SFPQ affect the formation of sense and antisense RNA foci and accumulation of

© 2021. Published by The Company of Biologists under the terms of the Creative Commons Attribution License (https://creativecommons.org/licenses/by/4.0/).

dipeptide repeat proteins (DPRs), and insist that modulation of SFPQ is a potential therapeutic approach to treat ALS/FTD with C9orf72 mutations. Though their hypothesis seems to be attractive, their results are not enough to validate this.

Comments for the author

1) The authors already reported that knockdown of SFPQ in C9orf72 mutation-positive patient fibroblasts reduced the number of GGGGCC RNA foci in JCS last year. In this paper, they showed that the number of antisense of RNA foci of GGGGCC repeats was also reduced by SFPQ knockdown, though SFPQ did not bind the antisense repeats in vitro. This needs more mechanistic explanation. Actually, the effect of SFPQ overexpression gave much smaller effect on the antisense RNA foci than that on the sense RNA foci. More detailed analysis for the rational reasoning of these would be required.

2) They showed that expression of DPRs was affected by SFPQ expression. They attribute this to the transcriptional regulation of GGGGCC repeat and hypothesized a negative regulation loop. Transcriptome analysis of SFPQ overexpression and knockdown cells with RNA-seq is required to validate their hypothesis.

First revision

Author response to reviewers' comments

Response to reviwers for Malnar and Rogelj

Reviewer 1 Comments for the author
This is an interesting study by Malnar and Rogelj, which examines the role of SFPQ in regulating RNA foci and dipeptide repeat proteins (DPRs) from C9orf72 mutations. The main conclusions are 1) SFPQ overexpression results in increased RNA foci and DPRs in HEK293s; 2) SFPQ KD results in reduced RNA foci and DPRs in HEK293s, patient-derived C9orf72 mutant fibroblasts and lymphoblasts. The authors therefore very reasonably conclude that SFPQ has a role in regulating c9 mutation. Overall, this study is well conceived and clearly articulated. Moreover, it is important for the field and I am in favour of publication subject to relatively minor revisions / clarifications:

1) For the RIP experiments, it should be noted much SFPQ protein in each pull down. Also G4C2-48 plasmid used, then G4C2-72 later on - could the change be justified in the text please.
Reply - Thank you for your comment on missing information about the amount of starting material used and the basis for different G4C2 constructs used in RNA pull-down experiments and cell transfection. To address the first comment, for RNA pull-down experiments we did not use purified SFPQ protein but rather mouse brain lysates. We used 400 mg of mouse brain tissue per each pull-down experiment. We added this information to Materials and Methods, RNA pull-down assay section, the addition is highlighted in yellow.

To address the second comment, we aimed for the longest possible G4C2 sequence to be used in all the aspects, thus, for RNA pull-down experiment and for transfection of the cells. The G4C2-48 was the longest construct we could produce with S1m aptamer on one side for the RNA pull-down experiment due to difficulties in cloning the complex GC-rich sequence. The information was added to Results: “SFPQ does not bind antisense RNA in vitro” section, the addition is highlighted in yellow. The G4C2-72 plasmid was used for cell transfection due to its successful translation to sense-derived DPRs in cells and it was previously published (Lee et al., 2013; Lee et al., 2020). We added this explanation to Results section: “SFPQ knockdown reduces RNA foci number and DPR expression in HEK cells”, the addition is highlighted in yellow.

© 2021. Published by The Company of Biologists under the terms of the Creative Commons Attribution License (https://creativecommons.org/licenses/by/4.0/).

2) Regarding SFPQ KD in HEKs, could the KD data be shown so that the efficiency of KD can be visualised.
Reply - Thank you for your comment regarding SFPQ KD in HEK cells. The SFPQ KD in HEK cells is presented in Figure 2, A with western blot of SFPQ and GAPDH and with graphical representation of SFPQ expression levels in SFPQ KD (shSFPQ) and control (scScramble) conditions. The western blots are also presented in the supplemental material (Figure S2). We hope this is a satisfactory presentation of SFPQ KD in HEKs.

3) The authors show antisense foci reduction, but no SFPQ association with antisense foci on RIP - It is of course plausible that the role of SFPQ in foci production doesn't necessitate direct interaction, but this should be discussed.
Reply - This comment is well appreciated. We add this point in the fourth paragraph in Discussion, added information is highlighted in yellow. In fact, we reorganized the third and fourth paragraph in Discussion to make it more coherent.

4) Could the authors clarify whether there was a decrease in PA or PR DPRs
Reply-This comment is very welcome. We addressed the expression of PA and PR DPRs in C9orf72 fibroblasts and C9orf72 lymphoblasts. However, we did not do so in the HEK cells, due to only sense-derived DPRs production. We hope we have now clarified this with the response to your first comment: "The G4C2-72 plasmid was used for cell transfection due to its successful translation to sense-derived DPRs in cells". We added this explanation to Results section: "SFPQ knockdown reduces RNA foci number and DPR expression in HEK cells", the addition is highlighted in yellow. The PA and PR expression levels were analysed in C9orf72 mutation-positive cells, as here PA and PR were produced from the endogenous expanded repeat RNA, which we did not achieve in the transfected HEK cells. Nevertheless, we find the information collected from the mutation-positive cells to be even of greater value and contribution to the research.

5) In the SFPQ KD in C9 mutant cells, SFPQ appears to form nuclear puncta, which possibly colocalise with antisense RNA foci. However, how can this be reconciled with no RIP association?
Reply - Thank you for the great comment. In this study we were interested in the direct interaction between SFPQ and sense and antisense RNA, which we have determined with RNA pull-down assay. We did not observe clear colocalization with antisense foci in the cells. Nevertheless, SFPQ is an abundant nuclear protein and some overlap with the antisense foci fluorescent signal is bound to be present. Furthermore, we do not exclude the possibility of antisense foci interacting with SFPQ-interacting proteins and therefore, to be present in the vicinity of SFPQ. Furthermore, antisense foci have been shown to colocalize with sense RNA foci (Zhang and Ashizawa, 2017). This could also lead to potential overlap between antisense RNA foci and SFPQ fluorescent signal in the cells due to SFPQ interaction with the sense foci.

6) In the Discussion, the following points should be considered:
-What role does sequestered SFPQ have in the stability of the RNA foci?
Reply - This comment is greatly appreciated. We added the missing information addressing this issue in the third paragraph of the Discussion. The additions are highlighted in yellow.

-Address lack of RIP interaction between SFPQ and anti-sense foci. Does this argue for different pathways of regulation of SFPQ upon anti or sense RNA foci.-Is the relationship between SFPQ and DPRs downstream to the interaction of the protein with the RNAs?
Reply - Thank you for this comment. We have addressed the lack of interaction between SFPQ and antisense foci in regard to your previous comment in the third and fourth paragraph of the Discussion. The additions are highlighted in yellow. We also addressed the possibility of SFPQ influencing DPRs downstream of the protein-RNA interaction in the fifth paragraph of the Discussion. The additions are highlighted in yellow.

-Do these data suggest a protective function as sequestered SFPQ would be unavailable to promote transcription of the c9 repeat RNAs and thereby DPR translation? Worth discussing further
Reply - This comment is very welcome. This possibility is addressed in the sixth paragraph of the Discussion, which we have modified for clarity.

© 2021. Published by The Company of Biologists under the terms of the Creative Commons Attribution License (https://creativecommons.org/licenses/by/4.0/).

Overall these experiments constitute a valid and robust approach to address role of SFPQ in levels of C9 associated species. The work is sound from an experimental perspective (power and appropriate methods used, over reliance on any one cell line is avoided through the use of fibroblasts, lymphoid cells and 293s). Cumulatively this study strengthens the cause for SFPQ protein as a player in the most common ALS type (C9orf72), and whilst future studies would include further interrogation of the function SFPQ plays in production of these RNAs, and how it's role differs between the sense and antisense species production, the current study adds important knowledge to the field.

Minor points
-The clockwise figure presentation is non-intuitive to follow.
Reply - Thank you for the valuable observation. We adapted the figures accordingly.

-The authors should clarify which RNA repeat did and did not bind to SFPQ in the text on line 105.
Reply - Thank you for pointing out the missing information. The information was added to Results: "SFPQ does not bind antisense RNA in vitro" section, the addition is highlighted in yellow.

-Please can the author explain why they have used a 48 repeat construct to look at SPFQ interaction in Figure 1 but then followed with a 72 repeat construct throughout the rest of the paper.
Reply - This comment is highly appreciated. We have addressed it in the previous comment section (comment 1) and added the missing information to Results: "SFPQ does not bind antisense RNA in vitro" section, and to Results: "SFPQ knockdown reduces RNA foci number and DPR expression in HEK cells" section. The additions are highlighted in yellow.

-The foci shown in figure 3C are difficult to see.
Reply - Thank you for your observation, we improved the intensity of foci in the mentioned figure.

-Please can the author clarify what they mean by 'biological repeats' and 'technical repeats' and thus clarifying how many times the experiment was independently conducted.
Reply - Thank you for the comment on insufficient information in regard to the number of independent experiment performance. The information was added to the Materials and Methods, Quantification and statistical analysis. The addition is highlighted in yellow. Information was also added to the Figure legends.

Reviewer 2 Advance summary and potential significance to field
The relevance of SFPQ expression and intracellular localization of C9orf72 is interesting issue to understand the pathological mechanism of ALS/FTD. In this manuscript, the authors showed that expression level of SFPQ affect the formation of sense and antisense RNA foci and accumulation of dipeptide repeat proteins (DPRs), and insist that modulation of SFPQ is a potential therapeutic approach to treat ALS/FTD with C9orf72 mutations. Though their hypothesis seems to be attractive, their results are not enough to validate this.

Reviewer 2 Comments for the author
1)The authors already reported that knockdown of SFPQ in C9orf72 mutation-positive patient fibroblasts reduced the number of GGGGCC RNA foci in JCS last year. In this paper, they showed that the number of antisense of RNA foci of GGGGCC repeats was also reduced by SFPQ knockdown, though SFPQ did not bind the antisense repeats in vitro. This needs more mechanistic explanation. Actually, the effect of SFPQ overexpression gave much smaller effect on the antisense RNA foci than that on the sense RNA foci. More detailed analysis for the rational reasoning of these would be required.
Reply - We are very grateful for this comment, which was also raised by Reviewer 1. Additions and modifications pertaining to this comment have been added to third and fourth paragraph of the Discussion. The additions are highlighted in yellow.

2)They showed that expression of DPRs was affected by SFPQ expression. They attribute this to the transcriptional regulation of GGGGCC repeat and hypothesized a negative regulation loop. Transcriptome analysis of SFPQ overexpression and knockdown cells with RNA-seq is required to validate their hypothesis.

© 2021. Published by The Company of Biologists under the terms of the Creative Commons Attribution License (https://creativecommons.org/licenses/by/4.0/).

Reply - With all due respect and apologies, we do not completely understand the relevance of the proposed RNAseq experiment.
As an RNA binding protein, SFPQ has already been shown to regulate RNA processing including transcription, splicing, transport and translation (Cosker et al., 2016; Hirose et al., 2014; Imamura et al., 2014; Kanai et al., 2004; Lee et al., 2015; Song et al., 2005; Takeuchi et al., 2018; Younas et al., 2020). This is the main drive for the question addressed in this paper – "Does SFPQ also regulate the repeat RNA foci and DPRs from C9orf72?". In Bajc Česnik et al. (Bajc Česnik et al., 2019) we showed that SFPQ KD reduces the level of sense foci and in this manuscript we expand our study to include overexpression of SFPQ, the effect of SFPQ levels also on antisense foci and DPRs, and addition of patient-derived lymphoblast cell lines that increases the disease relevance of the study. With this in mind, we believe that the proposed RNAseq experiment does not add to our question as this would provide a global view of the effect SFPQ on the transcriptome, which has already been extensively studied (Dong et al., 2005; Dong et al., 2007; Dong et al., 2011; Emili et al., 2002; Gordon et al., 2020; Hirose et al., 2014; Hosokawa et al., 2019; Kameoka et al., 2004; Lee et al., 2015; Mathur et al., 2001; Rosonina et al., 2005; Song et al., 2005; Stagsted et al., 2020; Takayama et al., 2017; Takeuchi et al., 2018).
We mentioned the negative feedback loop in the discussion section as one of the possible mechanisms arising from our observation in combination with published data. As explained above, this is not question/hypothesis of our paper. Our study is focused on the effect of SFPQ levels on sense and antisense repeat foci and, in our view, looking at the broad effect of SFPQ on the whole transcriptome would not add additional insight pertinent to our study.

Literature cited in the answers to reviewers:

Bajc Česnik, A., Darovic, S., Prpar Mihevc, S., Štalekar, M., Malnar, M., Motaln, H., Lee, Y.-B., Mazej, J., Pohleven, J., Grosch, M., et al. (2019). Nuclear RNA foci from C9ORF72 expansion mutation form paraspeckle-like bodies. J. Cell Sci. 132,.
Cosker, K. E., Fenstermacher, S. J., Pazyra-Murphy, M. F., Elliott, H. L. and Segal, R. A. (2016). The RNA-binding protein SFPQ orchestrates an RNA regulon to promote axon viability. Nat. Neurosci. 19, 690–696.
Dong, X., Shylnova, O., Challis, J. R. G. and Lye, S. J. (2005). Identification and characterization of the protein-associated splicing factor as a negative co-regulator of the progesterone receptor. J. Biol. Chem. 280, 13329–13340.
Dong, X., Sweet, J., Challis, J. R. G., Brown, T. and Lye, S. J. (2007). Transcriptional activity of androgen receptor is modulated by two RNA splicing factors, PSF and p54nrb. Mol. Cell. Biol. 27, 4863–4875.
Dong, L., Zhang, X., Fu, X., Zhang, X., Gao, X., Zhu, M., Wang, X., Yang, Z., Jensen, O. N., Saarikettu, J., et al. (2011). PTB-associated splicing factor (PSF) functions as a repressor of STAT6-mediated Ig epsilon gene transcription by recruitment of HDAC1. J. Biol. Chem. 286, 3451–3459.
Emili, A., Shales, M., McCracken, S., Xie, W., Tucker, P. W., Kobayashi, R., Blencowe, B. J. and Ingles, C. J. (2002). Splicing and transcription-associated proteins PSF and p54nrb/nonO bind to the RNA polymerase II CTD. RNA N. Y. N 8, 1102–1111.
Gordon, P. M., Hamid, F., Makeyev, E. V. and Houart, C. (2020). A conserved role for SFPQ in repression of pathogenic cryptic last exons. bioRxiv 2020.03.18.996827.
Hirose, T., Virnicchi, G., Tanigawa, A., Naganuma, T., Li, R., Kimura, H., Yokoi, T., Nakagawa, S., Bénard, M., Fox, A. H., et al. (2014). NEAT1 long noncoding RNA regulates transcription via protein sequestration within subnuclear bodies. Mol. Biol. Cell 25, 169–183.
Hosokawa, M., Takeuchi, A., Tanihata, J., Iida, K., Takeda, S. and Hagiwara, M. (2019). Loss of RNA-Binding Protein Sfpq Causes Long-Gene Transcriptopathy in Skeletal Muscle and Severe Muscle Mass Reduction with Metabolic Myopathy. iScience 13, 229–242.
Imamura, K., Imamachi, N., Akizuki, G., Kumakura, M., Kawaguchi, A., Nagata, K., Kato, A., Kawaguchi, Y., Sato, H., Yoneda, M., et al. (2014). Long noncoding RNA NEAT1-dependent SFPQ relocation from promoter region to paraspeckle mediates IL8 expression upon immune stimuli. Mol. Cell 53, 393–406.
Kameoka, S., Duque, P. and Konarska, M. M. (2004). p54nrb associates with the 5' splice site within large transcription/splicing complexes. EMBO J. 23, 1782–1791.
Kanai, Y., Dohmae, N. and Hirokawa, N. (2004). Kinesin Transports RNA: Isolation and Characterization of an RNA-Transporting Granule. Neuron 43, 513–525.
Lee, Y.-B., Chen, H.-J., Peres, J. N., Gomez-Deza, J., Attig, J., Stalekar, M., Troakes, C., Nishimura, A. L., Scotter, E. L., Vance, C., et al. (2013). Hexanucleotide repeats in ALS/FTD form

© 2021. Published by The Company of Biologists under the terms of the Creative Commons Attribution License (https://creativecommons.org/licenses/by/4.0/).

length-dependent RNA foci, sequester RNA binding proteins, and are neurotoxic. Cell Rep. 5, 1178–1186.
Lee, M., Sadowska, A., Bekere, I., Ho, D., Gully, B. S., Lu, Y., Iyer, K. S., Trewhella, J., Fox, A. H. and Bond, C. S. (2015). The structure of human SFPQ reveals a coiled-coil mediated polymer essential for functional aggregation in gene regulation. Nucleic Acids Res. 43, 3826–3840.
Lee, Y.-B., Baskaran, P., Gomez-Deza, J., Chen, H.-J., Nishimura, A. L., Smith, B. N., Troakes, C., Adachi, Y., Stepto, A., Petrucelli, L., et al. (2020). C9orf72 poly GA RAN-translated protein plays a key role in amyotrophic lateral sclerosis via aggregation and toxicity. Hum. Mol. Genet.
Mathur, M., Tucker, P. W. and Samuels, H. H. (2001). PSF is a novel corepressor that mediates its effect through Sin3A and the DNA binding domain of nuclear hormone receptors. Mol. Cell. Biol. 21, 2298–2311.
Rosonina, E., Ip, J. Y. Y., Calarco, J. A., Bakowski, M. A., Emili, A., McCracken, S., Tucker, P., Ingles, C. J. and Blencowe, B. J. (2005). Role for PSF in mediating transcriptional activator-dependent stimulation of pre-mRNA processing in vivo. Mol. Cell. Biol. 25, 6734–6746.
Song, X., Sun, Y. and Garen, A. (2005). Roles of PSF protein and VL30 RNA in reversible gene regulation. Proc. Natl. Acad. Sci. U. S. A. 102, 12189–12193.
Stagsted, L. V. W., O'Leary, E. T. and Hansen, T. B. (2020). The RNA-binding protein SFPQ preserves long-intron splicing and regulates circRNA biogenesis. bioRxiv 2020.09.04.280750.
Takayama, K., Suzuki, T., Fujimura, T., Yamada, Y., Takahashi, S., Homma, Y., Suzuki, Y. and Inoue, S. (2017). Dysregulation of spliceosome gene expression in advanced prostate cancer by RNA-binding protein PSF. Proc. Natl. Acad. Sci. 114, 10461–10466.
Takeuchi, A., Iida, K., Tsubota, T., Hosokawa, M., Denawa, M., Brown, J. B., Ninomiya, K., Ito, M., Kimura, H., Abe, T., et al. (2018). Loss of Sfpq Causes Long-Gene Transcriptopathy in the Brain. Cell Rep. 23, 1326–1341.
Younas, N., Zafar, S., Shafiq, M., Noor, A., Siegert, A., Arora, A. S., Galkin, A., Zafar, A., Schmitz, M., Stadelmann, C., et al. (2020). SFPQ and Tau: critical factors contributing to rapid progression of Alzheimer's disease. Acta Neuropathol. (Berl.) 140, 317–339.
Zhang, N. and Ashizawa, T. (2017). RNA toxicity and foci formation in microsatellite expansion diseases. Curr. Opin. Genet. Dev. 44, 17–29.

Second decision letter

MS ID#: JOCES/2020/256602

MS TITLE: SFPQ regulates the accumulation of RNA foci and dipeptide repeat proteins from the expanded repeat mutation in C9orf72

AUTHORS: Mirjana Malnar and Boris Rogelj
ARTICLE TYPE: Research Article

As you will see from their reports, the reviewers' recommendations are mixed. While referee #1 enthusiastically recommends publication, referee #2 remains negative. After carefully reading your study, I consider that your paper is acceptable for publication in Journal of Cell Science, pending standard ethics checks. Referee reports are appended below.

Reviewer 1

Advance summary and potential significance to field

I have summarised this in my previous review of the first version of this paper. I would like to congratulate these authors on an important manuscript in the field of ALS, which I feel will be well cited. I enthusiastically recommend this revised manuscript for publication

Comments for the author

These authors have thoughtfully addressed my concerns and I would like to congratulate these authors on an important manuscript in the field of ALS, which I feel will be well cited. I enthusiastically recommend this revised manuscript for publication

© 2021. Published by The Company of Biologists under the terms of the Creative Commons Attribution License (https://creativecommons.org/licenses/by/4.0/).

Reviewer 2

Advance summary and potential significance to field

The authors improved a part of Discussion but did not add any data to respond my requests.

Comments for the author

As I mentioned before, new information reported in this manuscript is too small.
The authors improved a part of Discussion but did not add any data to respond my requests.Their conclusion is too hypothetical and the improvement in the revised manuscript is quite limited. Therefore, I do not recomend this manuscript for publication in JCS.

© 2021. Published by The Company of Biologists under the terms of the Creative Commons Attribution License (https://creativecommons.org/licenses/by/4.0/).
